# Multidrug-Resistant *Campylobacter jejuni* Outbreak Linked to Puppy Exposure — United States, 2016–2018

**DOI:** 10.15585/mmwr.mm6737a3

**Published:** 2018-09-21

**Authors:** Martha P. Montgomery, Scott Robertson, Lia Koski, Ellen Salehi, Lauren M. Stevenson, Rachel Silver, Preethi Sundararaman, Amber Singh, Lavin A. Joseph, Mary Beth Weisner, Eric Brandt, Melanie Prarat, Rick Bokanyi, Jessica C. Chen, Jason P. Folster, Christy T. Bennett, Louise K. Francois Watkins, Rachael D. Aubert, Alvina Chu, Jennifer Jackson, Jason Blanton, Amber Ginn, Kirtana Ramadugu, Danielle Stanek, Jamie DeMent, Jing Cui, Yan Zhang, Colin Basler, Cindy R. Friedman, Aimee L. Geissler, Samuel J. Crowe, Natasha Dowell, Staci Dixon, Laura Whitlock, Ian Williams, Michael A. Jhung, Megin C. Nichols, Sietske de Fijter, Mark E. Laughlin

**Affiliations:** ^1^Ohio Department of Health; ^2^Epidemic Intelligence Service, CDC; ^3^Division of Foodborne, Waterborne, and Environmental Diseases, National Center for Emerging and Zoonotic Infectious Diseases, CDC; ^4^Oak Ridge Institute for Science and Education, Oak Ridge, Tennessee; ^5^Caitta, Inc., Herndon, Virginia; ^6^Atlanta Research and Education Foundation, Atlanta, Georgia; ^7^Ohio Department of Agriculture; ^8^IHRC, Inc., Atlanta, Georgia; ^9^Florida Department of Health in Orange County; ^10^Florida Department of Health.

*Campylobacter* causes an estimated 1.3 million diarrheal illnesses in the United States annually (*1*). In August 2017, the Florida Department of Health notified CDC of six *Campylobacter jejuni* infections linked to company A, a national pet store chain based in Ohio. CDC examined whole-genome sequencing (WGS) data and identified six isolates from company A puppies in Florida that were highly related to an isolate from a company A customer in Ohio. This information prompted a multistate investigation by local and state health and agriculture departments and CDC to identify the outbreak source and prevent additional illness. Health officials from six states visited pet stores to collect puppy fecal samples, antibiotic records, and traceback information. Nationally, 118 persons, including 29 pet store employees, in 18 states were identified with illness onset during January 5, 2016–February 4, 2018. In total, six pet store companies were linked to the outbreak. Outbreak isolates were resistant by antibiotic susceptibility testing to all antibiotics commonly used to treat *Campylobacter* infections, including macrolides and quinolones. Store record reviews revealed that among 149 investigated puppies, 142 (95%) received one or more courses of antibiotics, raising concern that antibiotic use might have led to development of resistance. Public health authorities issued infection prevention recommendations to affected pet stores and recommendations for testing puppies to veterinarians. This outbreak demonstrates that puppies can be a source of multidrug-resistant *Campylobacter* infections in humans, warranting a closer look at antimicrobial use in the commercial dog industry.

## Epidemiologic Investigation

Campylobacteriosis became a nationally notifiable condition in 2015, and many states routinely interview patients with campylobacteriosis.* For this investigation, a standardized, supplemental questionnaire was used by state and local health departments to collect dog exposure information from persons with *Campylobacter* infection who reported recent dog or pet store exposure during routine interview. A case definition relevant to this outbreak (Box) was developed to aid in case finding and characterization.

BOX Case definition for multidrug-resistant *Campylobacter jejuni* outbreak linked to puppy exposure — United States, 2016–2018*

**Confirmed case**

Campylobacteriosis in a patient with onset during January 1, 2016–February 28, 2018, who had either

- A clinical isolate closely related* to the outbreak strains by whole-genome sequencing (WGS), or
- Other laboratory evidence (culture or culture-independent diagnostic testing) of *Campylobacter* infection and worked in, visited, or had contact with a puppy from a pet store within 7 days before illness onset.

**Probable case**

An illness compatible with *Campylobacter* infection in a patient who had worked in, visited, or had contact with a puppy from a pet store within 7 days before illness onset, but without laboratory confirmation of *Campylobacter* infection.

**Exclusion criteria**

Exposure criteria met, but isolate unrelated to the outbreak strains by WGS.

* Relatedness of outbreak strains was determined by whole-genome multilocus sequence typing (wgMLST). Because no published wgMLST cutoff values exist, genetic relatedness was determined based on epidemiologic concordance and comparison with background *Campylobacter jejuni* isolates.

By February 28, 2018, a total of 118 persons meeting the case definition for *Campylobacter* infection, including 29 pet store employees, were reported from 18 states.^†^ Age was available for 115 persons and ranged from <1 year to 85 years (median = 26 years); 74 of 115 (63%) infected persons were female. Among 107 persons for whom hospitalization information was available, 26 (24%) were hospitalized; no deaths occurred. In total, 105 of 106 (99%) infected persons reported dog exposure, including 101 (95%) who had contact with a pet store puppy (Table). Eight patients reported buying or having contact with puppies from five pet store companies other than company A (companies B–F), indicating that puppies became infected with *Campylobacter* before reaching pet stores.

**TABLE:** Number of reported persons with *Campylobacter jejuni* infection during a multidrug-resistant outbreak, by reported puppy exposure — United States, 2016–2018*

Source	No. of infected persons reported
**Exposed to pet store puppy (n = 101)**	
Company A	92
Company B	3
Company C	2
Company D	1
Company E	1
Company F	1
Company unknown	1
**Purchased puppy from breeder**	3
**Adult dog exposure reported**	1
**No known dog exposure**	1
**Total reported**	**106**

* Excludes 12 patients for whom dog exposure questions were unknown.

State and local health and agriculture departments in four states (Kentucky, Ohio, Pennsylvania, and Wisconsin) visited 20 pet stores and collected antibiotic administration records for 154 puppies. Among 149 puppies with available information, 142 (95%) received one or more antibiotic courses before arriving or while at the store. Among 142 puppies that received antibiotics, treatment indication was available for 134 (94%); 78 (55%) treated puppies received antibiotics for prophylaxis only, 54 (38%) for prophylaxis and treatment, and two (1%) for treatment only. Median antibiotic treatment duration was 15 days (range = 2–67 days). Four antibiotics (metronidazole, sulfadimethoxine, doxycycline, and azithromycin) accounted for 81% of all antibiotics administered (Figure). Use of broad-spectrum antibiotics also was noted, including tetracyclines, quinolones, aminoglycosides, and chloramphenicol.

**FIGURE:**
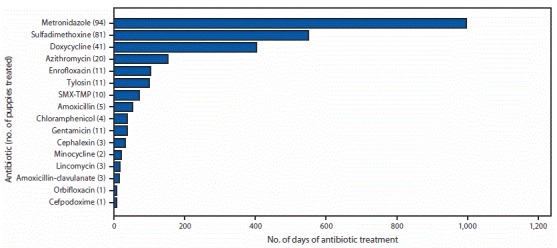
Number of days of antibiotics administered to 149 pet store puppies* assessed during a multidrug-resistant *Campylobacter jejuni* outbreak, by type of antibiotic — United States, 2016–2018 **Abbreviation:** SMX-TMP = sulfamethoxazole-trimethoprim. * Excludes five puppies with missing information on number of days treated.

## Laboratory Investigation

Stool specimens from infected persons or *Campylobacter* isolates were submitted to state public health laboratories. Health and agriculture officials from six states (Florida, Kansas, Kentucky, Ohio, Pennsylvania, and Wisconsin) visited 29 pet stores (27 company A and two company B) to collect puppy fecal samples. All company A and B stores in Ohio were visited, and a convenience sample of stores in other states was selected. Some states also requested fecal samples from patient households that had purchased puppies. Human stool specimens and puppy fecal samples underwent *Campylobacter* culture, and whole-genome multilocus sequence typing (wgMLST) was performed to compare genetic relatedness. Antibiotic susceptibility testing for nine antibiotics was performed by broth microdilution (Sensititer, Thermo Fisher Scientific) on selected isolates and interpreted using epidemiologic cutoff values established by the European Committee on Antimicrobial Susceptibility Testing. In this report, “resistant” refers to isolates with non–wild-type results (*2*). To explore pet food as a possible source of *Campylobacter* infection in puppies, dog food samples from company A and one person’s home were collected for culture.

*Campylobacter jejuni* isolates were obtained for 51 persons and 23 puppies. Outbreak isolates from 45 persons and 11 puppies grouped into three distinct clades by wgMLST. Six persons whose illnesses did not meet the case definition because their isolates were unrelated by wgMLST were excluded. Twelve puppy isolates were also unrelated to the outbreak by wgMLST. Two clades contained isolates from persons and puppies that were genetically related (≤32 alleles difference within each clade). The third clade contained six patient isolates that were related (≤30 alleles difference). Eighteen outbreak isolates (10 human and eight puppy) representing all three clades were selected for antibiotic susceptibility testing, and all were resistant to azithromycin, ciprofloxacin, clindamycin, erythromycin, nalidixic acid, telithromycin, and tetracycline. In addition, 16 of 18 isolates were resistant to gentamicin, and four of 18 were resistant to florfenicol. None of the cultures of 12 dog food samples yielded *Campylobacter*.

## Traceback Investigations

Records, including microchip identification numbers of puppies when available, were collected for puppies owned by infected persons and those sampled in stores. Microchips are implanted subcutaneously, usually before the puppy arrives at the store, and their corresponding identification numbers allowed investigators to trace puppies back to their breeders and distributors. Distributors are companies that purchase puppies wholesale from breeders and sell them to pet stores and other locations. Additional transport information was collected from stores when available. Practices identified during records review indicated that pet store puppies travel from breeders to distributors to stores by third-party transport companies. Information collected for eight puppies owned by infected persons and 20 puppies with fecal samples that were positive for *Campylobacter jejuni* traced back to 25 breeders and eight distributors. No single breeder, distributor, or transporter was identified as the infection source. However, potential for *Campylobacter* transmission among puppies exists because puppies from different breeders were commingled at distributors, during transport, and in stores.

## Public Health Response

CDC developed educational materials on campylobacteriosis prevention. CDC and states shared these with pet industry partners, including retail pet stores. Educational messages focused on handwashing, separating human eating areas from animal areas, and using personal protective equipment correctly, such as wearing gloves when cleaning cages in pet stores. CDC posted an outbreak advisory online, which included information for clinicians and veterinarians recommending culture and antibiotic susceptibility testing to guide antibiotic treatment decisions (*3*).

## Discussion

Epidemiologic, laboratory, and traceback evidence indicates that puppies sold through the commercial dog industry, an uncommon source of *Campylobacter* outbreaks, were the source of a multistate outbreak of multidrug-resistant *Campylobacter* infections. This evidence, combined with the prolonged nature of the outbreak and the potential for puppy commingling, indicates a potential for continued transmission of multidrug-resistant *Campylobacter* industrywide, including at breeders, distributors, transporters, and stores, and ultimately in customers' homes. Although the investigation is completed, the risk for multidrug-resistant *Campylobacter* transmission to employees and consumers continues.

Dog-associated *Campylobacter* outbreaks have been reported previously, but those outbreaks involved fewer illnesses, and the isolates were not multidrug-resistant (*4*–*6*). The investigation of this outbreak revealed widespread administration of multiple antibiotic classes, including all classes to which the outbreak *Campylobacter* strains were resistant. Hygiene and animal husbandry practices can reduce the need for antibiotics and decrease transmission of *Campylobacter* between animals and from animals to humans (*7*). Adherence to antibiotic stewardship practices in these settings might reduce the selection of highly drug-resistant *Campylobacter*. Implementation of antibiotic stewardship principles and practices in the commercial dog industry is needed.

Clinicians should consider that persons can acquire *Campylobacter* infections, including multidrug-resistant infections, from puppies. If antibiotics are indicated, consider stool culture and antibiotic susceptibility testing. Pet stores, commercial distributors, transporters, and breeders should ensure that existing biosecurity measures are sufficient to reduce ongoing risk for *Campylobacter* transmission between puppies and humans. Pet stores should provide employee and customer education and training on handwashing and provide employees with personal protective equipment when cleaning animal areas (*8*). Educational information^§^ that veterinarians and pet stores provide to pet owners could include information on reducing the risk for pathogen transmission. Finally, antibiotics should only be administered under veterinary supervision with a valid veterinary-client-patient relationship, consistent with existing stewardship principles.^¶^

**Summary**

**What is already known about this topic?**

Dogs, especially puppies, are a known source of sporadic *Campylobacter* infections in humans, but are uncommonly reported to cause outbreaks.

**What is added by this report?**

Investigation of a multistate, multidrug-resistant outbreak of *Campylobacter jejuni* infections implicated puppies from breeders and distributors sold through pet stores as the outbreak source. Outbreak strains were resistant to all antibiotics commonly used to treat *Campylobacter* infections.

**What are the implications for public health practice?**

Consumers, employees, and clinicians should be aware of the risk for disease transmission from puppies, including the possibility of exposure to multidrug-resistant pathogens. Greater adherence to implementation of antibiotic stewardship practices in the commercial dog industry might be needed.

## Footnotes

* https://wwwn.cdc.gov/nndss/conditions/campylobacteriosis/case-definition/2015/.

† Connecticut (two patients), Florida (20), Georgia (five), Illinois (11), Kansas (seven), Massachusetts (two), Maryland (five), Michigan (one), Missouri (two), New Hampshire (two), New York (two), Ohio (34), Oklahoma (one), Pennsylvania (six), Tennessee (two), Utah (four), Wisconsin (nine), and Wyoming (three).

§ https://www.cdc.gov/healthywater/hygiene/etiquette/around_animals.html.

¶ https://www.avma.org/KB/Policies/Pages/Antimicrobial-Stewardship-Definition-and-Core-Principles.aspx.
